# Association between the pig genome and its gut microbiota composition

**DOI:** 10.1038/s41598-019-45066-6

**Published:** 2019-06-19

**Authors:** Daniel Crespo-Piazuelo, Lourdes Migura-Garcia, Jordi Estellé, Lourdes Criado-Mesas, Manuel Revilla, Anna Castelló, María Muñoz, Juan M. García-Casco, Ana I. Fernández, Maria Ballester, Josep M. Folch

**Affiliations:** 1 Plant and Animal Genomics, Centre for Research in Agricultural Genomics (CRAG), CSIC-IRTA-UAB-UB Consortium, Bellaterra, Spain; 2 Departament de Ciència Animal i dels Aliments, Facultat de Veterinària, Universitat Autònoma de Barcelona (UAB), Bellaterra, Spain; 3 Departament de Genètica i Millora Animal, Institut de Recerca i Tecnologia Agroalimentàries (IRTA), Caldes de Montbui, Spain; 4 Génétique Animale et Biologie Intégrative (GABI), Institut National de la Recherche Agronomique (INRA), AgroParisTech, Université Paris-Saclay, Jouy-en-Josas, France; 5 Departamento de Mejora Genética Animal, Instituto Nacional de Investigación y Tecnología Agraria y Alimentaria (INIA), Madrid, Spain; 6 Centro I+D en Cerdo Ibérico INIA-Zafra, Zafra, Spain

**Keywords:** Microbial ecology, Metagenomics, Genetic markers, Genome-wide association studies

## Abstract

The gut microbiota has been evolving with its host along the time creating a symbiotic relationship. In this study, we assess the role of the host genome in the modulation of the microbiota composition in pigs. Gut microbiota compositions were estimated through sequencing the V3-V4 region of the 16S rRNA gene from rectal contents of 285 pigs. A total of 1,261 operational taxonomic units were obtained and grouped in 18 phyla and 101 genera. *Firmicutes* (45.36%) and *Bacteroidetes* (37.47%) were the two major phyla obtained, whereas at genus level *Prevotella* (7.03%) and *Treponema* (6.29%) were the most abundant. Pigs were also genotyped with a high-throughput method for 45,508 single nucleotide polymorphisms that covered the entire pig genome. Subsequently, genome-wide association studies were made among the genotypes of these pigs and their gut microbiota composition. A total of 52 single-nucleotide polymorphisms distributed in 17 regions along the pig genome were associated with the relative abundance of six genera; *Akkermansia*, *CF231*, *Phascolarctobacterium*, *Prevotella*, *SMB53*, and *Streptococcus*. Our results suggest 39 candidate genes that may be modulating the microbiota composition and manifest the association between host genome and gut microbiota in pigs.

## Introduction

The digestive tract of animals has been evolving along the time with symbiotic microorganisms. These microbes, mostly bacteria, have adapted to thrive in such conditions forming complex and vital interactions among them and their host^1,2^. The ecological community of these microorganisms is called microbiome, and the interactions with the host can be commensal, pathogenic or mutualistic^3^. In this scenario, mutualistic gut microbiota provides the host with beneficial functions that the host cannot perform, such as digesting complex polysaccharides, producing vitamins, and preventing colonization by pathogens^2,4^. Likewise, commensal gut populations modulate hosts’ immune responses which can modify the microbiota composition in order to maintain gut homeostasis^4^. Therefore, apart from the host genetics, the complexity of the interactions increases taking into account factors such as age, diet, environment, disease, or maternal seeding which are known to influence gut microbial communities^5^.

The intestinal epithelium acts as a barrier, protecting deeper tissues from bacterial entry^2^. Supporting this defence system, the gut epithelial surface is coated with a mucous layer formed by mucin glycoproteins^6,7^. While the small intestine has only one layer which is permeable to bacteria^6^, the mucous layer of the colon is structured in two parts: a dense inner layer firmly attached to the gut epithelium that minimizes bacterial-epithelial cell contact, and a loose outer layer that can be broken down by commensal bacteria^7^. In this outer mucous layer, the metabolites produced by these bacteria interact with the host stimulating the innate and adaptive immune responses^2^. For instance, host innate immunity can select for a species-specific microbiota using microbicidal proteins^8^. However, the host also has mechanisms to tolerate the metabolites from non-pathogenic bacteria^2^, just as certain bacteria trigger the host immune system for self-benefit^9^.

In these recent years, high-throughput sequencing technologies have greatly improved the study of bacterial populations without performing microbial cultures. The microbial 16S rRNA gene sequencing is commonly used to estimate the microbiota composition, while the shotgun sequencing of DNA fragments isolated after shearing faecal or other samples is used for the metagenome (all the microbial collective genomes) characterization^10^. Recently, whole-metagenome sequencing has been used to obtain the reference gene catalogue of the pig gut microbiome^11^. This study revealed that the reference catalogue of the porcine gut microbiome shared more non-redundant genes between human and pig than human and mouse^11^, suggesting pig as a better animal model than mouse because of their similarity with humans. Both species are omnivores and have monogastric digestive tracts which are analogous in anatomy, immunology and physiology^12^.

The heritability of the microbial genera composition of the pig gut has been reported to range from low to high values^13,14^. Accordingly, host genetics has been suggested as an important factor in the determination of gut microbial composition^15^. However, there are limited studies measuring the contribution of inter-individual variability modulating the bacterial communities and the effect of host polymorphisms on the establishment of the microbiota^16^. In this context, genome-wide association studies (GWAS), which have been widely used to analyse a plethora of complex traits, are now being used to study the link between the host and its microbiota composition^15,16^. With this approach, Blekhman *et al*.^17^ were the first to describe in humans the relationship between the abundance of *Bifidobacterium* and the single-nucleotide polymorphisms (SNPs) close to the lactase gene. In this case, lactase non-persistent recessive individuals who drink milk cannot break down lactose and thus, *Bifidobacterium* thrives using this available sugar^18^.

Conversely, host genetics appeared to have a minor impact in the microbiota compared with age, diet or the environment^19^. It is not surprising, since conditions are difficult to standardize between individuals. In this regard, production pigs represent a perfect model to measure the effect of host genetics in shaping the microbiota due to their similar diet and environmental factors during their whole rearing cycle, but the relationship between the pig genome and its gut microbiota composition has not yet been fully described^20^.

The objective of this study was to identify genomic regions that influence the gut microbiota composition through host-microbiota associations in pigs. For this purpose, the 16S rRNA gene was sequenced from rectal contents of 288 pigs genotyped with a high-throughput method.

## Materials and Methods

### Ethics approval

All animal manipulations were performed according to the regulations of the Spanish Policy for Animal Protection RD53/2013, which meets the European Union Directive 2010/63/EU about the protection of animals used in experimentation. Pigs were slaughter in a commercial abattoir following national and institutional guidelines for Good Experimental Practices.

### Animal material

A total of 288 healthy commercial F1 crossbred pigs (Duroc × Iberian) were used in this study. All animals were maintained in the same farm under intensive conditions and feeding was *ad libitum* with a barley- and wheat-based commercial diet. Pigs with an average weight of 138.8 kg (SD = 11.46 kg) were slaughtered in a commercial abattoir in four distinct days. Samples of rectal content and *Longissimus dorsi* muscle were snap-frozen in liquid nitrogen and later stored at −80 °C.

### Microbial DNA extraction and 16S rRNA gene sequencing

For each one of the 288 samples, the DNA of 0.2 g of rectal content was extracted with PowerFecal kit (MoBio Laboratories, Carlsbad, CA, USA) following the manufacturer’s recommendations. DNA purity and concentration were measured through a ND-1000 spectrophotometer (NanoDrop Technologies, Wilmington, DE, USA). The amplification of the V3-V4 region of the 16S rRNA gene was performed following the recommendations of the 16S *Metagenomic Sequencing Library Preparation* guide (Illumina, San Diego, CA, USA). Full description of primer sequences and methods used can be accessed at Supplementary Information S1. All the 288 amplicon pooled libraries were sequenced in three runs of a MiSeq (Illumina, San Diego, CA, USA) instrument in the Sequencing Service of the FISABIO (*Fundació per al Foment de la Investigació Sanitària i Biomèdica de la Comunitat Valenciana*, Valencia, Spain) using the MiSeq Reagent Kit v3 (600-cycle format, 2 × 300 bp paired-end reads). A mean of 104,115 reads for each sample was obtained (17.991 Gb in total), ranging from 34,186 to 218,360 reads, except for one outlier that was discarded because it had 1,758,983 reads.

### Taxonomy classification and diversity studies of the gut samples

Bioinformatics analysis were performed in QIIME v.1.9.1^21^ by using the QIIME’s subsampled open-reference operational taxonomic unit (OTU) calling approach and following the recommendations of Rideout *et al*.^22^. In brief, the *join_paired_ends.py* function in QIIME was used to merge the forward and reverse reads contained in the fastq files of the remaining 287 samples. The quality control and the filtering process was made pursuant to the considerations provided by Bokulich *et al*.^23^. Therefore, the *split_libraries_fastq.py* command was used to demultiplex and filter (at Phred ≥ Q20) the fastq sequence data. After this step, OTUs were identified by using the *pick_open_reference_otus.py* function with a subsampled percentage of 10% (s = 0.1). Subsequently, chimera detection was carried on in QIIME with BLAST^24^ and OTUs were taxonomically annotated employing the Greengenes 13.8 database^25^. At this point, two samples did not satisfy the quality filters and were discarded. Thus, for the remaining 285 samples, a dataset containing 1,294 OTUs was obtained after filtering out singletons and OTUs representing less than 0.005% of the total number of annotated reads^23^. From this dataset, 33 OTUs had missing taxonomic ranks and were discarded. Finally, 1,261 OTUs in the 285 samples were considered for further analysis (Supplementary Table S1).

The 1,261 OTUs were grouped in 18 phyla and 101 genera through the *tax_glom* method within the phyloseq package^26^ in R (www.r-project.org). Besides, genera that belonged to a higher taxonomy rank but lacked the genus information were merged and marked as unspecified (g__unsp).

The analyses of α and β-diversities in the 285 samples were carried on with the vegan R package^27^, and the non-metric multidimensional scaling (NMDS) plot was performed using phyloseq^26^ and ggplot2^28^. For the α-diversity study, the Shannon index was employed, whereas the β-diversity study was represented using the Whittaker index. Additionally, the dissimilarity between pairs of samples was estimated with the NMDS method using the Bray-Curtis dissimilarity^29^.

### Host DNA extraction and SNP genotyping

Pig genomic DNA was extracted from the *Longissimus dorsi* muscle of all the 288 samples using the standard phenol-chloroform method^30^. The DNA concentration and purity was measured with a ND-1000 spectrophotometer (NanoDrop) afterwards.

A total of 288 pigs were genotyped with the GeneSeek Genomic Profiler (GGP) Porcine HD v1 (70 K) array (Illumina, San Diego, CA, USA) using the Infinium HD Assay Ultra protocol (Illumina). Genotypes were obtained with the GenomeStudio software (2011.1 version, Illumina) and filtered with the PLINK software^31^ (1.90b5 version). Further analyses were conducted using only SNPs that mapped in the *Sscrofa11.1* assembly, with a minor allele frequency (MAF) >5% and missing genotypes <5%, retaining a total of 45,508 SNPs.

### GWAS analysis

For the 285 samples, GWAS between the microbiota composition at genus level and the 45,508 genotyped SNPs were made. Samples were normalized in percentages based on the number of annotated reads per sample (relative abundance). To avoid errors caused by low abundant genera, GWAS were performed only in genera that comprised more than the 0.5% of the total annotated reads and were present in more than the 90% of the samples. In addition, genera marked as unspecified were excluded from the GWAS analysis. Therefore, GWAS were performed in 18 of the 101 genera found.

For the GWAS analysis, the following univariate linear mixed model was applied using the GEMMA software^32^ (0.96 version):

$${{\rm{y}}}_{{\rm{ijkl}}}={{\rm{Sex}}}_{{\rm{i}}}+{{\rm{Batch}}}_{{\rm{j}}}+{{\rm{u}}}_{{\rm{k}}}+{{\rm{\delta }}}_{{\rm{k}}}{{\rm{a}}}_{{\rm{l}}}+{{\rm{e}}}_{{\rm{ijkl}}},$$

where y_ijkl_ indicates the vector of phenotypic observations in the k^th^ individual; sex (two categories) and batch (4 categories) are fixed effects; u_k_ is the infinitesimal genetic effect considered as random and distributed as N(0, Kσ_u_), where K is the numerator of the kinship matrix; δ_k_ is a −1, 0, +1 indicator variable depending on the k^th^ individual genotype for the l^th^ SNP; a_l_ represents the additive effect associated with the l^th^ SNP; and e_ijkl_ is the residual.

The false discovery rate (FDR) method developed by Benjamini and Hochberg^33^ was applied for multiple test correction using the *p.adjust* function incorporated in R. The cut-off for considering a SNP as significant was set at FDR ≤ 0.1. Two significant SNPs were grouped inside the same interval if the distance between them was less than 2 Mb.

### Gene annotation and functional prediction

The associated regions in the pig genome were annotated at 1 Mb on each side of the previously defined intervals. The genes contained in these regions were extracted using the BioMart tool^34^ from the Ensembl project (www.ensembl.org; release 92) using the *Sscrofa11.1* reference assembly. In addition, the functional consequences of the significant SNPs were predicted through the Variant Effect Predictor tool^35^ from the Ensembl project (release 92).

## Results and Discussion

### Microbiota composition and diversity

**Figure 1:**
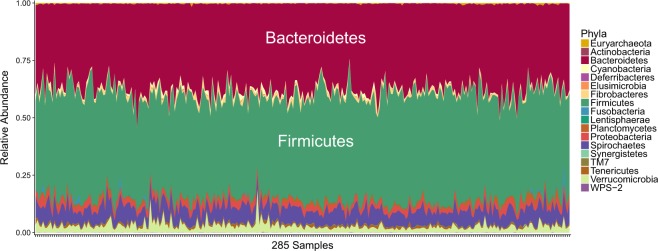
Stacked area plot of OTUs grouped by phyla for the 285 pig rectal samples.

A mean of 104,115 reads per sample were obtained with a MiSeq (Illumina) after sequencing the V3-V4 region of the 16S rRNA gene from rectal contents of 288 pigs. A total of 1,261 OTUs which were grouped in 18 phyla and 101 genera were found in the 285 samples that fulfilled the quality criteria. At phylum level, *Firmicutes* (45.36%) and *Bacteroidetes* (37.47%) were the more abundant (Fig. 1 and Supplementary Table S2). In accordance with the literature^36,37^, *Firmicutes* and *Bacteroidetes* are usually the most dominant phyla found in colon and faeces of pigs. The most abundant genera, not marked as unspecified, were *Prevotella* (7.03%) and *Treponema* (6.29%) (Supplementary Table S3). Accordingly, *Prevotella* spp. are frequently found as one of the most abundant genus in the lower intestine and faeces^36,37^. However, comparisons between different studies should be made with caution, since differences in microbiota composition are conditional on the different sets of primers used in the analysis, breeds (host genetic background), age of the animals at sampling time, and environmental factors such as dietary composition^14,38^.

**Figure 2:**
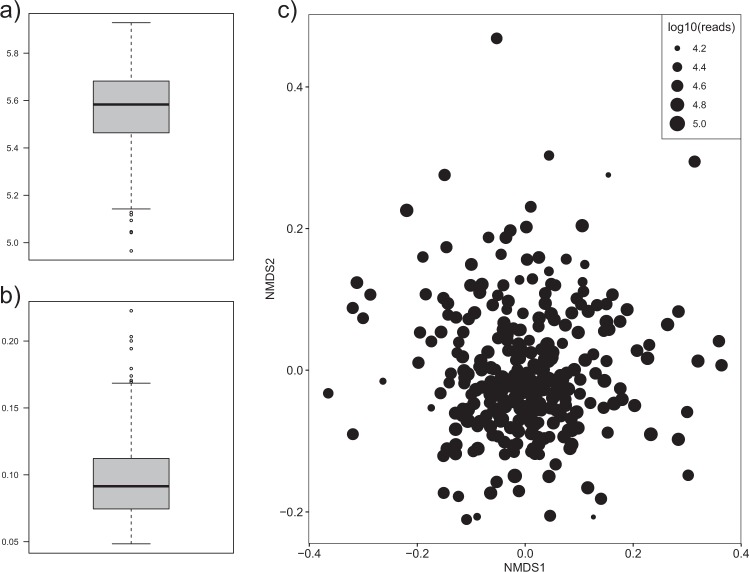
Plots showing the diversities and dissimilarities measured using the 1,261 OTUs found in rectal contents of 285 pigs. (**a**) Boxplot of the Shannon α-diversity. (**b**) Boxplot of the Whittaker β-diversity calculated through the Bray-Curtis dissimilarity. (**c**) Non-metric multidimensional scaling (NMDS) plot based on Bray-Curtis dissimilarities. The size of the dot is proportional to the total number of annotated reads in each sample.

To obtain a measure for the number of different OTUs and their relative abundance within each of the 285 samples, the community α-diversity was calculated through the Shannon index (Fig. 2a). The mean of the α-diversity was 5.55, ranging from 4.97 to 5.93. It is not surprising, since the distal part of the pig gut usually has a higher α-diversity than the rest of the intestine^37^. In addition, the β-diversity was used to measure the differences between samples through the Whittaker index (Fig. 2b) obtaining a mean distance to the centroid of 0.10. Lastly, a NMDS plot was performed to observe the dissimilarities between samples employing Bray-Curtis dissimilarity (Fig. 2c). The β-diversity was closer to 0, indicating that the global microbiota composition was quite similar along the 285 samples. Furthermore, this low β-diversity was expected, since the pigs from our study have been subjected to the same diet and environmental factors during their whole rearing cycle and this uniformity is reinforced by the absence of clustering in the NMDS plot. This way, overall diversity results reinforce the appropriateness of the model to measure the effect of host genetics in shaping the microbiota.

### GWAS results

**Figure 3:**
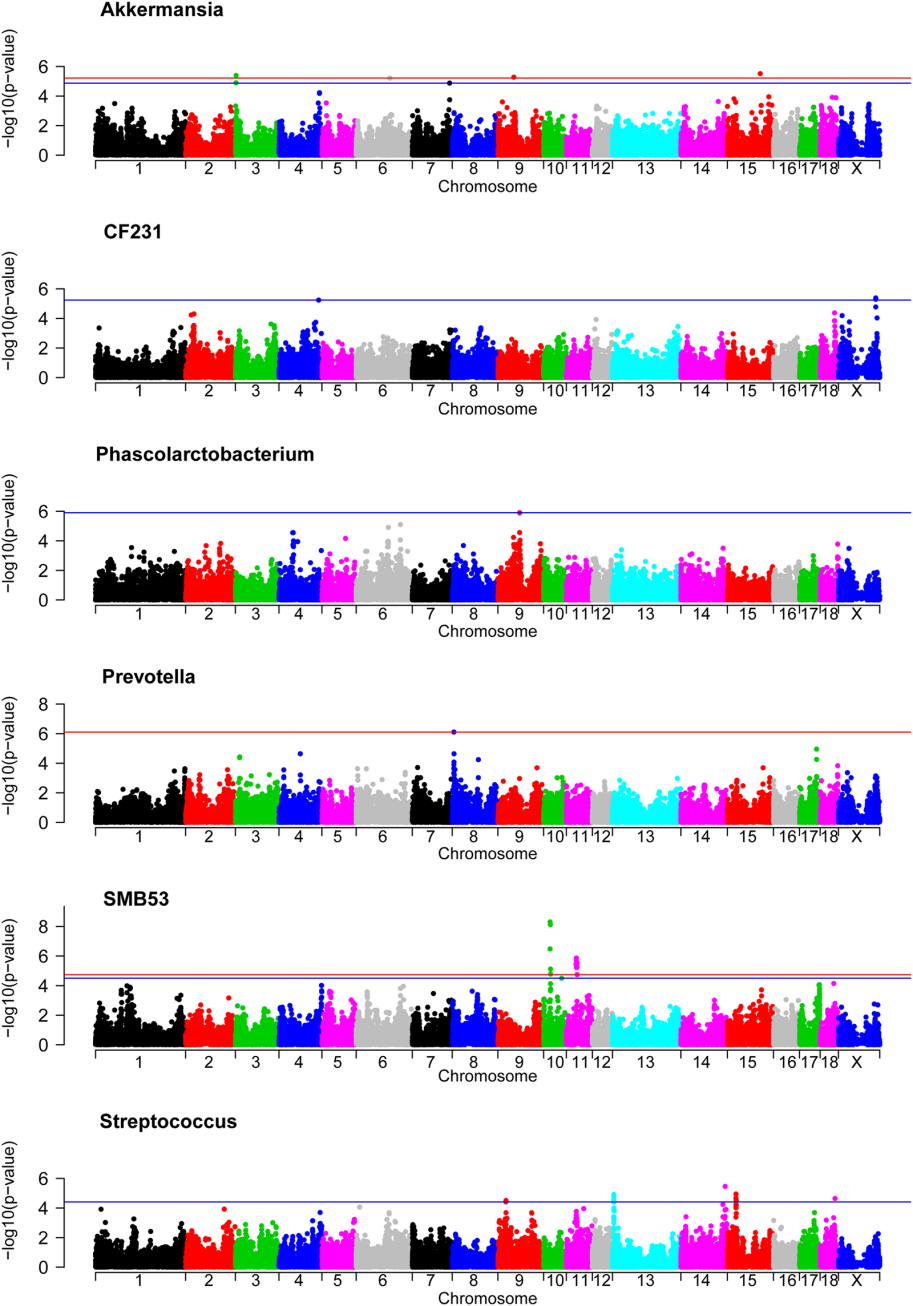
GWAS plot for the relative abundance of the following genera: *Akkermansia*, *CF231*, *Phascolarctobacterium*, *Prevotella*, *SMB53*, and *Streptococcus*. The red lines indicate those SNPs that are below the genome-wide significance threshold (FDR ≤ 0.05), while the blue lines indicate those SNPs that are below genome-wide significance threshold (FDR ≤ 0.1).

GWAS were performed using 45,508 SNPs genotyped in 285 animals and the relative abundance of 18 genera; *Akkermansia*, *Bacteroides*, *CF231*, *Coprococcus*, *Fibrobacter*, *Lactobacillus*, *Oscillospira*, *Parabacteroides*, *Paraprevotellaceae Prevotella*, *Phascolarctobacterium*, *Prevotella*, *RFN20*, *Ruminococcus*, *SMB53*, *Sphaerochaeta*, *Streptococcus*, *Treponema* and *YRC22*. A total of 52 significant SNPs were distributed in 17 regions along the following *Sus scrofa* chromosomes (SSC): SSC3, SSC4, SSC6, SSC7, SSC8, SSC9, SSC10, SSC11, SSC13, SSC14, SSC15, SSC18 and SSCX (Supplementary Table S4). Significant association signals (FDR ≤ 0.1) were found in six out of the 18 GWAS for the following genera: *Akkermansia*, *CF231*, *Phascolarctobacterium*, *Prevotella*, *SMB53* and *Streptococcus* (Fig. 3 and Table 1). No shared associated regions were found for the abundances of these six genera, albeit some of them belong to the same phyla. *CF231* and *Prevotella* are genera of the *Bacteroidetes* phylum. Within the *Firmicutes* phylum, *Phascolarctobacterium* and *SMB53* are members of the *Clostridiales* order, and *Streptococcus* is a member of the *Lactobacillales* order. Hence, our results suggest an association between chromosomal regions along the pig genome and abundance of certain bacteria genera. In the following sections, the candidate genes mapped in the genomic regions associated with the genus relative abundance of *Akkermansia*, *CF231*, *Phascolarctobacterium*, *Prevotella*, *SMB53* and *Streptococcus* are discussed in detail. The list of candidate genes is summarized in Table 1.

**Table 1:** Significant genomic regions in the pig genome associated with the relative composition of genera and the candidate genes found within.

Region	Genus	Chr.^a^	Position in Mb Start - End	No. SNPs^b^	Most significant SNP	Effect (%)	*p*-value	FDR	Candidate genes
A1	*Akkermansia*	3	0.89–3.04	3	rs81335357; rs81246645	7.72	4.04 × 10^−6^	4.58 × 10^−2^	*CARD11*; *CHST12*
A2	*Akkermansia*	6	101.46–103.46	1	rs81390429	25.85	6.06 × 10^−6^	4.58 × 10^−2^	*TGIF1*
A3	*Akkermansia*	7	112.61–114.61	1	rs325604118	6.56	1.33 × 10^−5^	7.55 × 10^−2^	*LGMN*; *CHGA*
A4	*Akkermansia*	9	47.53–49.57	2	rs81410866; rs81410881	8.78	5.25 × 10^−6^	4.58 × 10^−2^	*ssc-mir-125b-1*; *ssc-mir-100*; *SORL1*
A5	*Akkermansia*	15	99.10–101.10	1	rs80982646	7.94	3.04 × 10^−6^	4.58 × 10^−2^	*SLC39A10*
B1	*CF231*	4	119.91–121.91	1	rs319005051	7.07	5.72 × 10^−6^	6.48 × 10^−2^	
B2	*CF231*	X	112.48–114.50	3	rs329229283	6.34	4.10 × 10^−6^	6.48 × 10^−2^	*FGF13*; *ATP11C*
C1	*Phascolarctobacterium*	9	65.33–67.33	1	rs81223434	7.73	1.25 × 10^−6^	5.68 × 10^−2^	*SLC45A3*; *RAB7B*; *RAB29*; *NUCKS1*; *IKBKE*; *MAPKAPK2*
D1	*Prevotella*	8	3.81–5.81	1	rs326174858	10.88	7.79 × 10^−7^	3.53 × 10^−2^	*CYTL1*; *WFS1*; *MAN2B2*
E1	*SMB53*	10	18.51–22.05	5	rs344136854	14.25	4.92 × 10^−9^	1.68 × 10^−4^	*CAPN8*; *CAPN2*; SUSD4; *DENND1B*; *PTPRC*; ssc-mir-181b-1; ssc-mir-181a-1
E2	*SMB53*	10	53.89–55.89	1	rs341165563	8.87	3.15 × 10^−5^	7.14 × 10^−2^	*MALRD1*
E3	*SMB53*	11	28.22–33.50	14	rs80835110	10.42	1.37 × 10^−6^	1.47 × 10^−2^	*PCDH17*
F1	*Streptococcus*	9	23.45–25.66	3	rs319168851	6.15	3.11 × 10^−5^	9.41 × 10^−2^	*FAT3*
F2	*Streptococcus*	13	2.97–5.15	4	rs81310237	11.88	1.20 × 10^−5^	9.41 × 10^−2^	*PLCL2; GALNT15*; *RFTN1*
F3	*Streptococcus*	14	133.80–135.80	1	rs337448241	10.51	3.46 × 10^−6^	9.41 × 10^−2^	*CTBP2*; *UROS*
F4	*Streptococcus*	15	25.15–27.87	9	rs331341379	8.72	1.12 × 10^−5^	9.41 × 10^−2^	*ERCC3*; *BIN1*; *MAP3K2*
F5	*Streptococcus*	18	44.25–46.25	1	rs334064749	7.7	2.26 × 10^−5^	9.41 × 10^−2^	*ssc-mir-196b-1*

^a^Chromosome.

^b^Number of significant SNPs found in the region (FDR ≤ 0.1).

#### *Akkermansia*

The relative abundance of *Akkermansia*, a genus of the *Verrucomicrobia* phylum, was significantly associated with polymorphisms in five chromosomic regions: SSC3, SSC6, SSC7, SSC9, and SSC15 (Table 1). Within the SSC3 region (1.03–3.04 Mb), two candidate genes have been proposed, caspase recruitment domain family member 11 (*CARD11*) and carbohydrate sulfotransferase 12 (*CHST12*). CARD11 is necessary for T helper 17 cells differentiation which are involved in the adaptive immune system and protect the body against extracellular bacteria^39^. The *CHST12* gene is required for glycosaminoglycan biosynthesis^40^. Glycosaminoglycans are also called mucopolysaccharides and are often found in the mucin layer together with glycans and sialic acid^41^. The most common species of the *Verrucomicrobia* phylum found in the gut, *Akkermansia muciniphila*, colonizes the mucus layer and it is a known mucin degrader^42^. The regulation of host genes related to glycosaminoglycans biosynthesis probably has a direct effect in the occurrence of mucin degrading bacteria. Studying further this candidate gene may help to select a genetic variant that enriches the presence of *A. muciniphila*, since this species is beneficial to the host by restoring gut barrier function and helps reducing obesity^43^. In SSC6 (102.46 Mb), the only significant SNP (rs81390429, *p*-value = 6.06 × 10^−6^) explained a 25% of the variance in the abundance of the *Akkermansia* genus. The candidate gene found in this SSC6 region, *TGIF1* (TGFB induced factor homeobox 1), encodes for a protein that contributes to the adaptive immunity favouring the response of T follicular helper cells^44^. Additionally, two candidate genes were proposed for the *Akkermansia* spp. abundance in the SSC7 region (112.61–114.61 Mb): *CHGA* (chromogranin A) and *LGMN* (legumain). In humans, faecal levels of CHGA were associated with 61 different bacterial species including *A. muciniphila*, which was negatively associated with CHGA^45^. CHGA plays a role in the innate immunity with its antimicrobial activity against bacteria^46^, whereas LGMN is a cysteine protease that also has antimicrobial activity, as well as it is involved in the antigen-presenting process and Toll-like receptors (TLRs) activation^47^. Thus, variations in the *CHGA* or *LGMN* genes should be affecting the microbiota composition based on the bacterial resistance to their antimicrobial activity. Inside the SSC9 region (47.53–49.57 Mb), there were three candidate genes, two microRNAs genes, *ssc-mir-125b-1* and *ssc-mir-100*, and the sortilin related receptor 1 (*SORL1*) gene. Both microRNAs are involved with the adaptive immune system: *miR-125b-1* inhibits B cell differentiation^48^, while *miR-100* inhibits T cell proliferation and differentiation^49^. Therefore, polymorphisms in these miRNAs genes may affect the targeting of these miRNAs or their expression, which might be associated with the abundance of *Akkermansia* spp. However, the two significant SNPs (rs81410866 and rs81410881, *p*-value = 5.25 × 10^−6^) of the SSC9 region were located in intronic regions of the *SORL1* gene. SORL1 is an endocytic receptor that might be affecting gut microbiota composition as it has been associated with obesity^50^, and pancreatic and biliary tract cancer in humans^51^. Finally, a significant SNP (rs80982646, *p*-value = 3.04 × 10^−6^) located at 100.1 Mb in SSC15 was also associated with the relative abundance of *Akkermansia* spp. In this region, we identified the *SLC39A10* (solute carrier family 39 member 10) gene which appears to be a good candidate to modulate the presence of *Akkermansia* spp., since positively regulates B cell receptor signalling pathway^52^. Hence, in accordance with our results, germ-free mice colonized with *A. muciniphila* showed an overexpression in genes related with the antigen presentation pathway and B and T cell maturation, implying its possible role as host immune system modulator^53^.

#### *CF231*

The relative abundance of the *CF231* genus (a member of the *Paraprevotellaceae* family) was associated to genetic variations in two regions along the pig genome in SSC4 and SSCX (Table 1). While no candidate genes were found in SSC4 at 120.91 Mb, the SSCX region (112.48–114.50 Mb) contained the ATPase phospholipid transporting 11 C (*ATP11C*) and the fibroblast growth factor 13 (*FGF13*) genes. The ATP11C protein is involved in B cell differentiation past the pro-B cell stage, thus, defects in ATP11C led to a lower number of B cells and an impairment in their differentiation^54^. Changes in the *ATP11C* gene may cause species-specific tolerance through the adaptive immune system and transport. Additionally, ATP11C is also involved in the metabolism of cholestatic bile acids^55^. Intestinal content of cholesterol has the potential to shape the gut microbiome^56^ and the *CF231* genus might be affected by the expression of these genes, since bile acids are catabolites of cholesterol. Interestingly, an enrichment of the *CF231* genus has been detected in experiments with high fat diet-induced hypercholesterolemic rats treated with cholesterol-lowering drugs^57^. On the other hand, the three significant SNPs of the SSCX region were located in an intron of the other candidate gene, *FGF13* (Supplementary Table S4). Although the biological role of the *FGF13* gene is not clear, it may be involved in the repair of the intestinal epithelial damage affecting microbiota composition. This function has been reported in another member of its gene family, *FGF2* (fibroblast growth factor 2)^58^.

#### *Phascolarctobacterium*

Six candidate genes found inside the SSC9 region (65.33–67.33 Mb) may be associated with the relative abundance of *Phascolarctobacterium* spp. (Table 1). *Phascolarctobacterium* is a Gram-negative genus commonly found in human faeces able to produce short chain fatty acids (SCFAs)^59^. SCFAs are absorbed and serve as a source of energy by colonocytes and peripheral tissue or can be use as substrates for lipogenesis, gluconeogenesis or regulation of cholesterol synthesis in the liver^60^. Interestingly, one of the candidate genes that could be modulating the abundance of *Phascolarctobacterium* spp. was the *SLC45A3* (solute carrier family 45 member 3). SLC45A3 is involved in the positive regulation of fatty acid biosynthetic process^61^. There were also two other candidate genes within SSC9 which encode GTPases that are members of the RAS oncogene family (*RAB7B* and *RAB29*). Under the induction of the lipopolysaccharides present in the Gram-negative cell wall, RAB7B promotes the degradation of toll like receptor 4 (TLR4) impairing the innate immune response by reducing the sensitivity of macrophages to lipopolysaccharides signalling^62^. Therefore, RAB7B may play an important role in the development of tolerance to Gram-negative commensal bacteria such as *Phascolarctobacterium* spp. The other GTPase, RAB29, is involved in bacterial toxin transport and is able to discriminate between *Salmonella enterica* serovars^63^. The positive regulation of insulin receptor signalling pathway by the *NUCKS1* (nuclear casein kinase and cyclin dependent kinase substrate 1) gene^64^ located in this SSC9 region may also be modulating the abundance of the *Phascolarctobacterium* genus, since it has been described an enrichment of this genus in diabetic animal models treated with prebiotics to alleviate glucose intolerance^60^. Additionally, the two remaining candidate genes, *IKBKE* (inhibitor of nuclear factor kappa B kinase subunit epsilon) and *MAPKAPK2* (mitogen-activated protein kinase-activated protein kinase 2) might be associated with the microbiota composition because of their relationship with the immune system. IKBKE inhibits T cell responses^65^ and MAPKAPK2 regulates interleukin 10^66^ which is crucial to maintain the gut homeostasis^4^.

#### *Prevotella*

Studies performed in humans have associated the presence of the *Prevotella* genus with a high intake of complex fibres in the diet^67^. In our animal material, *Prevotella* spp. represented 7.03% of the total composition at genus level (Supplementary Table S3). In this case, only the *rs326174858* SNP located at 4.81 Mb in SSC8 was significantly associated (*p*-value = 7.79 × 10^−7^) with the abundance of *Prevotella* spp. (Table 1). Three candidate genes were found in this SSC8 region, cytokine like 1 (*CYTL1*), wolframin ER transmembrane glycoprotein (*WFS1*), and mannosidase alpha class 2B member 2 (*MAN2B2*). *CYTL1* codes for a protein capable of chemoattracting macrophages and its activity is sensitive to *Bordetella pertussis* toxin^68^. A defect in the second gene, *WFS1*, produces insulin insufficiency, causing diabetes via pancreatic β cells failures^69^. Therefore, diabetic individuals would reduce glucose uptake in the gut epithelium^70^. In this sense, glucose might be more available for some bacteria species, producing changes in the overall microbiota composition. In accordance with this hypothesis, the abundance of *Prevotella* spp. was reduced in diabetic children when compared to healthy ones^71^. The encoded protein of the last candidate gene, *MAN2B2*, is implicated in the degradation of glycans^72^. Glycans are excreted into the intestine, including those in dietary plants, animal-derived, cartilage and tissue (glycosaminoglycans and N-linked glycans), and endogenous glycans from host mucus (O-linked glycans)^73^. The *Prevotella* genus contributes to the degradation of mucin and plant-based carbohydrates^74^ and, therefore, it seems plausible that variations in a gene involved in the degradation of glycans could modulate the presence of *Prevotella* spp.

#### *SMB53*

The *SMB53* genus sequences found in swine compost were closely related with *Clostridium glycolicum*^75^. The abundance of the *SMB53* genus in our pig rectal samples accounted for 1.19% of the total number of annotated reads (Supplementary Table S3), and presented three significant associated regions, two in SSC10 and one in SSC11 (Table 1). The first region of SSC10 (18.51–22.05 Mb) showed the most significant SNP (rs344136854, *p*-value = 4.92 × 10^−9^). Seven candidate genes have been identified inside this SSC10 region: calpain 2 (*CAPN2*) and 8 (*CAPN8*); sushi domain containing 4 (*SUSD4*); DENN domain containing 1B (*DENND1B*); protein tyrosine phosphatase, receptor type C (*PTPRC*); *ssc-mir-181a-1* and *ssc-mir-181b-1*. Calpains are a family of proteases that are able to perform various cellular functions depending on changes in intracellular Ca^2+^ levels^76^. For instance, an increase in Ca^2+^ in the intestinal porcine endothelial cells due to the *Clostridium perfringens* β-toxin triggers the calpain activation leading to intestinal cell death^77^. Therefore, polymorphisms in the *CAPN2* or *CAPN8* genes may confer resistance to *Clostridium* spp. avoiding endothelial cell death and so, increasing *Clostridia* abundance in the gut. Two of the significant SNPs of this SSC10 region were located in an intron of the *SUSD4* gene, whereas other significant SNP was also located in an intron of the *DENND1B* gene (Supplementary Table S4). *SUSD4*, *DENND1B* and *PTPRC* are genes related with the immune system: SUSD4 inhibits the complement system^78^, DENND1B is a regulator of the T cell receptor signalling^79^, and PTPRC is necessary for antigen receptor mediated signalling in lymphocytes^80^. The last two candidate genes in this first SSC10 region were both microRNAs from the *miR-181* family. The depletion of *miR-181* causes a lack of Natural Killer T cells in the thymus as well as defects in T and B cells development^81^. In the second SSC10 region (54.89 Mb), the only significant SNP (rs341165563, *p*-value = 3.15 × 10^−5^) was located in an intron of the *MALRD1* (MAM and LDL receptor class A domain containing 1) gene (Supplementary Table S4). This candidate gene is involved in bile acid synthesis regulation and is able to modify the gut microbiota^82^. Khan *et al*.^57^ demonstrated an increase in the relative abundance of the *SMB53* genus in hypercholesterolemic rats treated with cholesterol-lowering drugs. Thus, further studies are needed to evaluate the modulation of the *SMB53* genus by the MALRD1 negative regulation of bile acid biosynthetic process. Additionally, the *SMB53* genus belongs to the *Clostridiaceae* family. Most members of this family have the capacity to consume gut mucus- and plant-derived saccharides like glucose^83^. Interestingly, recent studies performed by Horie *et al*.^84^ have detected an enrichment of *SMB53* in caecum of mice suffering type 2 diabetes, suggesting a possible role of this genus in the disease. The last region, in SSC11 (28.22–33.5 Mb), was comprised of 14 SNPs but, despite being the longest region observed (5.3 Mb), only one candidate gene (protocadherin 17, *PCDH17*) was proposed. Remarkably, the most significant SNP (rs80835110, *p*-value = 1.37 × 10^−6^) was located in an intron of *PCDH17* (Supplementary Table S4). PCDH17 may play a role in the colon similar to protocadherin 1 (PCDH1), acting as a physical barrier in the airway epithelial cells^85^.

#### *Streptococcus*

There are five regions within the pig genome associated to the presence of *Streptococcus* spp., SSC9, SSC13, SSC14, SSC15, and SSC18 (Table 1). In the SSC9 region (23.45–25.66 Mb), the protein encoded by the FAT atypical cadherin 3 (*FAT3*) gene may be forming epithelial junctions that can be broken down by *Streptococcus* spp.^86^. In the SSC13 region (2.97–5.15 Mb), three candidate genes were found: phospholipase C like 2 (*PLCL2*), polypeptide N-acetylgalactosaminyltransferase 5 (*GALNT15*), and raftlin, lipid raft linker 1 (*RFTN1*). Four significant SNPs were located in intronic regions of the *PLCL2* gene (Supplementary Table S4). PLCL2 increases the thresholds of B cell activation^87^ and so, it may modulate the tolerance of the adaptive immune system. On the other hand, GALNT15 belongs to a family of proteins that are able to produce O-linked glycosylation in the mucin^88^ and hence, the variations on the *GALNT15* gene might affect some mucin dwellers like *Streptococcus* spp.^89^. Additionally, it is also interesting to highlight a possible link between the *RFTN1* gene, involved in the formation and/or maintenance of lipid rafts^90^, and the abundance of the *Streptococcus* genus. The lipid rafts are microdomains located in the membrane surface of the cell that play an important role in cellular signaling and membrane trafficking of T and B lymphocytes^90,91^. Furthermore, lipid rafts are also mediators of innate immune recognition of bacteria^92^. The possible association of RFTN1 with the abundance of *Streptococci* needs further attention, since some species of *Streptococcus* are known to hijack these lipid rafts to enter the host cell causing disease^93^. Two candidate genes were found in the SSC14 region (133.8–135.8 Mb): *CTBP2* (C-terminal binding protein 2) and *UROS* (uroporphyrinogen III synthase). The *CTBP2* gene was associated in pigs with a susceptibility to develop a bacterial respiratory disease^94^. The *UROS* gene is involved in the metabolism of porphyrins including heme and uroporphyrinogen III biosynthetic processes^95^. Iron in mammals is incorporated into heme; an essential component of the hemoglobin, which can be acquired by bacterial pathogens as a nutritional iron source. Several *Streptococci* species that are pathogenic to humans and animals, namely *S. pyogenes*, *S. pneumoniae* and *S. suis*, contain cell wall heme-binding proteins that allow them to scavenge heme from host’s hemoglobin as a source of iron acquisition^96,97^. Additionally, the group B *Streptococci* are able to respire in the presence of heme, enhancing resistance to oxidative stress and improving their survival^98^. Our results suggest that the *UROS* gene may modulate the presence of *Streptococcus* spp. making these animals more susceptible to *Streptococci* colonization. A total of three candidate genes were identified in the SSC15 region (25.15–27.87 Mb): *ERCC3* (ERCC excision repair 3, TFIIH core complex helicase subunit), *BIN1* (bridging integrator 1), and *MAP3K2* (mitogen-activated protein kinase kinase kinase 2). The *ERCC3* gene expression was downregulated in human gastric cells after the infection with *Helicobacter pylori*^99^. In the same direction as the aforementioned *GALNT15* gene, the *BIN1* gene might modulate the abundance of mucin dweller bacteria like *Streptococcus* spp. because of the attenuation of *BIN1* favours the intestinal barrier function^100^. The protein encoded by the last candidate gene of this SSC15 region, *MAP3K2*, activates the toll like receptor 9 (TLR9) that recognizes CpG oligodeoxynucleotide motif in bacteria^101^. Finally, the last significant region (45.25 Mb) in SSC18 contained one microRNA, *ssc-mir-196b-1*, that was found upregulated in the duodenum of piglets that were resistant to *Escherichia coli* infection^102^.

## Conclusion

This report identifies associations between the pig genome and the relative abundance of six genera (*Akkermansia*, *CF231*, *Phascolarctobacterium*, *Prevotella*, *SMB53* and *Streptococcus*). Most of the candidate genes found in the 17 associated regions of the pig genome encode for proteins that are involved in the host defence system, including the immune system, physical barriers such as the mucin layer or cell junctions, whereas other proteins participate in the metabolism of mucopolysaccharides or bile acids. Our results confirm the importance of host genomics in the modulation of the microbiota composition. However, the associations found in this study could be specific of our population, as the associated polymorphisms found may not be segregating in other populations and the gut microbiota is affected by different factors such as breed, age and diet. Therefore, further studies are warranted in different populations to determine which genetic combinations favour the enrichment of beneficial bacteria, providing the individual with the best intestinal health to avoid the entrance of potential pathogens.

## Supplementary information

Supplementary Legends.

Supplementary Information S1.

Supplementary Table S1.

Supplementary Table S2.

Supplementary Table S3.

Supplementary Table S4.

## Data Availability

The raw sequencing data generated by this study were deposited in the NCBI Sequence Read Archive (SRA) under BioProject accession number PRJNA540380.
