# Supplementary material for: Association between the pig genome and its gut microbiota composition
Source: Sci Rep. 2019 Jun 19;9:8791. doi: 10.1038/s41598-019-45066-6 (PMC6584621; doi:10.1038/s41598-019-45066-6)
Supplement: Supplementary file 1 — Supplementary Legends. [file 41598_2019_45066_MOESM1_ESM.pdf]

Association between the pig genome and its gut microbiota composition

Daniel Crespo-Piazuelo[1,2,*], Lourdes Migura-Garcia[3], Jordi Estellé[4], Lourdes Criado-Mesas[1], Manuel Revilla[1,2], Anna Castelló[1,2], María Muñoz[5,6], Juan M García-Casco[5,6], Ana I Fernández[5], Maria Ballester[3], and Josep M Folch[1,2]

[1]Plant and Animal Genomics, Centre for Research in Agricultural Genomics (CRAG), CSIC-IRTA-UAB-UB Consortium, Bellaterra, Spain

[2]Departament de Ciència Animal i dels Aliments, Facultat de Veterinària, Universitat Autònoma de Barcelona (UAB), Bellaterra, Spain

[3]Departament de Genètica i Millora Animal, Institut de Recerca i Tecnologia Agroalimentàries (IRTA), Caldes de Montbui, Spain

[4]Génétique Animale et Biologie Intégrative (GABI), Institut National de la Recherche Agronomique (INRA), AgroParisTech, Université Paris-Saclay, Jouy-en-Josas, France

[5]Departamento de Mejora Genética Animal, Instituto Nacional de Investigación y Tecnología Agraria y Alimentaria (INIA), Madrid, Spain

[6]Centro I+D en Cerdo Ibérico INIA-Zafra, Zafra, Spain

*E-mail: daniel.crespo@cragenomica.es

Supplementary Information

Supplementary Information S1. Full description of the 16S rRNA gene amplification and sequencing.

Supplementary Table S1. Table containing the read counts for the 1,261 OTUs found in rectal contents of 285 pigs after the filtering processes. Taxonomic ranks are included in the first seven columns.

Supplementary Table S2. Means for the relative abundance of the 18 phyla found in rectal contents of 285 pigs. %_Presence indicates the percentage of the pigs where these phyla were found.

Supplementary Table S3. Means for the relative abundance of the 101 genera found in rectal contents of 285 pigs. %_Presence indicates the percentage of the pigs where these genera were found.

Supplementary Table S4. Description of the 52 significant genera-associated SNPs and their predicted consequences with the Variant Effect Predictor tool[35] (Ensembl release 92). MAF indicates the minor allele frequency.
