# Supplementary material for: Association between the pig genome and its gut microbiota composition
Source: Sci Rep. 2019 Jun 19;9:8791. doi: 10.1038/s41598-019-45066-6 (PMC6584621; doi:10.1038/s41598-019-45066-6)
Supplement: Supplementary file 2 — Supplementary Information S1. [file 41598_2019_45066_MOESM2_ESM.pdf]

Association between the pig genome and its gut microbiota composition

Daniel Crespo-Piazuelo[1,2,*], Lourdes Migura-Garcia[3], Jordi Estellé[4], Lourdes Criado-Mesas[1], Manuel Revilla[1,2], Anna Castelló[1,2], María Muñoz[5,6], Juan M García-Casco[5,6], Ana I Fernández[5], Maria Ballester[3], and Josep M Folch[1,2]

[1]Plant and Animal Genomics, Centre for Research in Agricultural Genomics (CRAG), CSIC-IRTA-UAB-UB Consortium, Bellaterra, Spain

[2]Departament de Ciència Animal i dels Aliments, Facultat de Veterinària, Universitat Autònoma de Barcelona (UAB), Bellaterra, Spain

[3]Departament de Genètica i Millora Animal, Institut de Recerca i Tecnologia Agroalimentàries (IRTA), Caldes de Montbui, Spain

[4]Génétique Animale et Biologie Intégrative (GABI), Institut National de la Recherche Agronomique (INRA), AgroParisTech, Université Paris-Saclay, Jouy-en-Josas, France

[5]Departamento de Mejora Genética Animal, Instituto Nacional de Investigación y Tecnología Agraria y Alimentaria (INIA), Madrid, Spain

[6]Centro I+D en Cerdo Ibérico INIA-Zafra, Zafra, Spain

*E-mail: daniel.crespo@cragenomica.es

16S rRNA gene amplification and sequencing

The following primers were used to amplify the V3-V4 region of the 16S rRNA gene as stated by Klindworth *et al*. (2013) and the *16S Metagenomic Sequencing Library Preparation* guide (Illumina, San Diego, CA, USA): Forward = 5' TCG TCG GCA GCG TCA GAT GTG TAT AAG AGA CAG CCT ACG GGN GGC WGC AG 3' and Reverse = 5' GTC TCG TGG GCT CGG AGA TGT GTA TAA GAG ACA GGA CTA CHV GGG TAT CTA ATC 3'. Each one of the 288 PCR reactions were carried out individually in a total volume of 25 µL utilizing 12.5 ng of microbial DNA, 12.5 µL of 2× KAPA HiFi HotStart ReadyMix (Kapa Biosystems, Wilmington, MA, USA) and 5 µL of each primer (1 µM) with the following protocol: 95ºC for 3 min, 25 cycles of three steps (95ºC for 30 s, 55ºC for 30 s and 72ºC for 30 s) and 72ºC for 5 min. The amplicon expected size (~550 bp) was verified via agarose gel electrophoresis. Then, the PCR product clean-up was performed with AMPure XP beads (Beckman Coulter, Beverly, MA, USA). After this step, the dual indices were attached with the Nextera XT Index Kit and another PCR clean-up round was performed with AMPure XP beads afterwards.

References

Klindworth, A. *et al*. Evaluation of general 16S ribosomal RNA gene PCR primers for classical and next-generation sequencing-based diversity studies. *Nucleic. Acids. Res.* 2013; **41**: 1–11.
